# Complement activation in atypical hemolytic uremic syndrome and scleroderma renal crisis: a critical analysis of pathophysiology

**DOI:** 10.1590/2175-8239-JBN-3807

**Published:** 2018-05-07

**Authors:** Roman Zuckerman, Arif Asif, Eric J. Costanzo, Tushar Vachharajani

**Affiliations:** 1 Jersey Shore University Medical Center, Seton Hall-Hackensack-Meridian School of Medicine, Neptune, NJ, USA.; 2 W.G. (Bill) Hefner VA Medical Center, Salisbury, North Carolina, USA.

**Keywords:** Complement Activation, Scleroderma, Systemic, Acute Kidney Injury, Ativação de Complemento, Esclerodermia, Sistêmica, Lesão renal aguda

## Abstract

Scleroderma is an autoimmune disease that affects multiple systems. While pathophysiologic mechanisms governing the development of scleroderma are relatively poorly understood, advances in our understanding of the complement system are clarifying the role of complement pathways in the development of atypical hemolytic uremic syndrome and scleroderma renal crisis. The abundant similarities in their presentation as well as the clinical course are raising the possibility of a common underlying pathogenesis. Recent reports are emphasizing that complement pathways appear to be the unifying link. This article reviews the role of complement system in the development of atypical hemolytic uremic syndrome and scleroderma renal crisis, and calls for heightened awareness to the development of thrombotic angiopathy in patients with scleroderma.

## RESUMO

A esclerodermia é uma doença autoimune que afeta múltiplos sistemas. Embora os mecanismos fisiopatológicos que regem o desenvolvimento da esclerodermia sejam relativamente pouco compreendidos, os avanços em nossa compreensão do sistema do complemento estão esclarecendo o papel das vias do complemento no desenvolvimento da síndrome urêmica hemolítica atípica e da crise renal da esclerodermia. As abundantes semelhanças em sua apresentação, bem como o curso clínico, estão aumentando a possibilidade de uma patogênese subjacente comum. Relatórios recentes estão enfatizando que as vias de complemento parecem ser o link unificador. Este artigo analisa o papel do sistema do complemento no desenvolvimento da síndrome urêmica hemolítica atípica e da crise renal na esclerodermia, e exige maior conscientização para com o desenvolvimento da angiopatia trombótica em pacientes com esclerodermia.

## INTRODUCTION

Systemic sclerosis (SSc) or scleroderma is an autoimmune heterogeneous disease involving multiple systems and is classically divided into limited, diffuse, and overlap forms of the disease.^1^ Three distinct pathophysiologic mechanisms continue to dominate the disease process. These include, 1) a vascular injury leading to release of vasoconstrictor mediators and tissue hypoxia, 2) immunogenicity culminating in production of antibodies, and 3) fibroblast dysfunction resulting in increased deposition of extracellular matrix.^2^^-^ 16 Some features and manifestations of SSc are skin thickening, Raynaud phenomenon, digital ulcers, pulmonary arterial hypertension (PAH), interstitial lung disease (ILD), and renal disease. While PAH and ILD are important causes of death in patients with SSc, recent reports are emphasizing the development of thrombotic microangiopathy (TMA) with its ensuing mortality (Table 1).^4^^-^ 11

**Table 1:** Scleroderma renal crisis patients presenting with thrombotic microangiopathy

Reference #	Age/gender	Plasma therapy	Eculizumab	ESRD	Death	Diagnosis rendered
49	48 F	Yes	No	Yes	No	HUS/TTP
58	35 F	Yes	No	No	No	TTP
52	73 M	No	No	Yes	No	HUS
53	48 F	No	No	Yes	Yes	HUS
59	31 F	Yes	No	Yes	Yes	TTP
47	61 F	No	No	Yes	No	HUS
61	32 F	Yes	No	Yes	Yes	TTP
55	58 M	Yes	No	Yes	Yes	HUS
41	46 F	Yes	Yes	No	No	aHUS
40	28 F	No	Yes	Yes	Yes	aHUS

### SCLERODERMA AND THROMBOTIC MICROANGIOPATHY

TMAs are a group of disorders characterized by widespread microvascular thrombosis, thrombocytopenia, and microangiopathic hemolytic anemia (MAHA).^17^ TMAs are traditionally classified into thrombotic thrombocytopenic purpura (TTP), atypical hemolytic uremic syndrome (aHUS), and Shiga toxin-associated HUS. In general, Shiga toxin-associated HUS occurs secondary to infection with *Escherichia coli* serotypes 0157:H7, 0111:H8, 0103:H2, 0123, 026, or others that produce Shiga-like toxin. This form of TMA is not associated with SSc and is beyond the scope of this paper. However, both aHUS and TTP have been reported with SSc.^4^^,^ 11 ^,^ 18 ^-^ 21 TTP is caused by the deficiency of ADAMTS13 while aHUS results from an uncontrolled activation of the alternative pathway of the complement system. 22 ^-^ 23 Histologically, on renal biopsy, aHUS is indistinguishable from HUS caused by toxin-producing bacteria or TTP. In acute cases, thrombi are identified within the glomerular capillaries, arterioles as well as arteries and are accompanied by endothelial cell swelling or denudation. Over time, there is thickening of glomerular capillary walls (double contour), loosening of mesangial architecture (mesangiolysis) caused by accumulation of plasma proteins fibrin and fibrinogen, and the emergence of membranoproliferative pattern of injury.^24^

Dysregulation of the alternative pathway of the complement system leading to its uncontrolled activation results in aHUS.^22^^-^ 27 The complement system is one of the first defenses of the immune system to be mobilized against a pathogen. Complement proteins are produced in the liver and are present in blood, lymph, and extracellular fluids. The three pathways of the complement system (classic, lectin, and alternative) produce protease complexes termed C3 and C5 convertases that cleave C3 and C5 respectively, eventually leading to the membrane-attack complex.^23^ C3 hydrolysis in plasma initiates the alternative pathway, leading to the deposition of C3b onto practically all plasma-exposed surfaces.^23^ Complement activation is controlled by various membrane-anchored and fluid-phase regulators.^28^ Factors B, D, and C3 participate in the generation of the alternative pathway C3 convertase (C3bBb), which is stabilized by factor P (properdin). C3 cleavage by the C3 convertases and subsequent C5 cleavage by the C5 convertases results in the formation of C5a and C5b. The latter participates in the assembly of the membrane attack complex (MAC; C5b-9, soluble terminal complement complex (sTCC)). MAC mediates target cell activation, injury or lysis in a dose-dependent manner. The alternative pathway of the complement system is constitutively active and its activity is kept in check by several soluble and membrane-bound complement regulators.^24^ Common soluble complement regulatory proteins include factor I, factor H, and C4-binding protein.^22^^-^ 24 Similarly, complement regulators also exist on the surface of cells and include membrane cofactor protein (MCP), decay accelerating factor (DAF), and complement regulator 1 (CR1) etc.^24^ Mutations of these regulatory proteins lead to an uncontrolled activation of the complement system causing endothelial injury and resulting in aHUS. Indeed, genetic abnormalities in complement system proteins have been documented both in the familial and sporadic forms of aHUS.^25^ Multiple mutations in factors regulating the alternative complement pathway are found in 40-60% of patients with aHUS.^26^^,^ 27

In simple terms, three elements are needed to have a high index of suspicion for aHUS. These include thrombocytopenia, microangiopathic hemolytic anemia, and target organ injury.^22^ Thrombocytopenia with a platelet count < 150,000/µL or a 25% decline from the baseline, hemoglobin below 10g/dL, intravascular hemolysis with elevated LDH and reduced haptoglobin, and schistocytes on peripheral smear all add to the diagnosis. Complement C3 level might be reduced with normal concentrations of C4, as well as elevated C5a and C5b-9 complex.^24^ Recent studies have demonstrated that the levels of membrane bound C5b-9 complex deposits on human microvascular endothelial cells are increased in patients with aHUS and can be used as a marker for activation of the biological processes.^29^^,^ 30 However, these findings are neither sensitive nor specific for diagnosis and of limited prognostic value, with reduced levels of C3 found in only in 30-50% of patients with certain complement mutations.^25^ End-organ damage (kidney, brain, heart, gastrointestinal tract) also adds to the diagnosis. Finally, ADAMTS13 helps in excluding the diagnosis of TTP. While important, at present, genetic testing to establish the diagnosis of aHUS is not mandatory, as only 50-60% of the genetic mutations are currently known.^25^^-^ 27

### THE ROLE OF COMPLEMENT IN SCLERODERMA

Can the complement system be involved in the pathogenesis of SSc? Complement proteins have been studied in relation to SSc for over 30 years.^31^^-^ 37 Studies have pointed out the activation of the classical pathway of the complement system in patients with diffuse SSc.^31^^-^ 34 Recent studies have demonstrated hypocomplementemia in patients with SSc overlap disease.^35^^-^ 37 A gene screening study of anti-RNA polymerase III (ARA+) patients who developed scleroderma renal crisis (SRC) showed a strong association with the complement system.^15^ Batal et al. clearly demonstrated C4d deposits (a classical complement pathway degradation product) in patients with SRC, especially in those with worse outcomes (death, or requiring dialysis or transplant).^38^ Very recently, a Swedish study demonstrated that patients with SRC had lower levels of C3 and factor B secondary to over-activation of the alternative pathway.^39^ However, the serum levels of sTCC were lower in subjects with SRC.^39^ This is a confounding finding given that one would expect to find increased levels during the initial stages of the acute phase of the renal crisis. One reason for the discrepancy might be the actual timing and stage of the acute phase relative to the time of sample collection. Additionally, the investigators did not measure the amount of MAC deposition on surface of cells. A possible explanation of low sTCC might also be its quick removal from the circulation and prompt deposition at the tissue level. However, one case report of a patient with SRC did demonstrate an elevated serum level of sTCC (along with decreased levels of both C3 and C4).^40^ This patient was treated with eculizumab therapy demonstrating hematological remission. Unfortunately, the patient died 8 weeks later, secondary to new onset heart failure.^40^ Another patient with scleroderma overlap syndrome (positive for PM-Scl antibodies) presented with acute renal failure, thrombocytopenia, and microangiopathic hemolytic anemia.^41^ She was initially treated with plasmapheresis for a presumed diagnosis of TTP. Because of a complete lack of improvement, a diagnosis of aHUS was considered. Plasmapheresis was discontinued and the patient was treated with eculizumab with complete resolution of the thrombocytopenia and the microangiopathic hemolytic anemia, and significant recovery of renal function.^41^

It is worth exploring the induction of thrombosis/microthrombosis involving endothelial cells, adhesion molecules, as well as prothrombinase. C5a is a potent trigger of inflammation responsible for expression of tissue factor (TF) on endothelial cells, monocytes, and neutrophils. TF in turn allows the formation of prothrombinase complex. Further activation of coagulation factor II (prothrombin) generates small amount of thrombin (IIa). Thrombin induces platelet activation, adhesion, and aggregation. Platelets are involved in complement activation by cleaving C3 into its components (C3a and C3Bb). Blocking the cleavage of C5 into C5a and C5b by eculizumab prevents the formation of the MAC and stops the amplification loop.^42^

Endothelial cells appear to be the common platform for both aHUS and scleroderma. These cells are continuously exposed to the actions of biologically active products of the complement system.^43^ Whether it is the uncontrolled activation of the alternative pathway (due to mutations of the regulatory proteins) or the activation of the classic pathway (due to auto-antibodies in scleroderma), the generation of C5b-C9 terminal complex deposited on endothelial cells is directly involved in activation of human microvascular endothelial cell 1 (HMEC-1) through increased expression of soluble vascular cell adhesion molecule-1 (sVCAM-1) and tissue factor (TF).^22^^-^ 24 ^,^ 31 ^-^ 34 ^,^ 40 ^,^ 41 Injury to HMEC-1 is demonstrated by release of thrombomodulin from damaged cells.^44^ Additionally, it induces secretion of multimers of von Willebrand factor and stimulates prothrombinase. Direct platelet activation is further triggered by cellular retraction and exposed underlying prothrombotic matrix, resulting in microthrombosis.^45^ These pathological processes ultimately lead to target organ injury (Table 1).^46^^-^ 62

## CONCLUSION

The abundant similarities in the presentation as well as clinical course of scleroderma renal crisis and aHUS raise a question of whether there is a common pathogenesis involved. Complement pathways appear to be the unifying link. Activation and injury to the endothelium due to persistent stimulation by the complement system creates a pathological loop responsible for thrombotic microangiopathy and target organ injury. Several reports have shown that eculizumab was effective in blocking the sTCC in patients with scleroderma renal crisis who presented with symptoms resembling aHUS. Future studies involving patients with aHUS are needed in order to elucidate the pathogenesis of scleroderma presenting with thrombotic microangiopathy.
